# Conceptualizing Emotion Regulation and Coregulation as Family-Level Phenomena

**DOI:** 10.1007/s10567-022-00378-4

**Published:** 2022-01-30

**Authors:** Blair Paley, Nastassia J. Hajal

**Affiliations:** Department of Psychiatry and Biobehavioral Sciences, Jane and Terry Semel Institute for Neuroscience and Human Behavior, University of California, Los Angeles, USA

**Keywords:** Emotion regulation, Coregulation, Family secure base, Prevention, Intervention

## Abstract

The ability to regulate one’s emotions is foundational for healthy development and functioning in a multitude of domains, whereas difficulties in emotional regulation are recognized as a risk factor for a range of adverse outcomes in childhood, adolescence, and adulthood. Caregivers play a key role in cultivating the development of emotion regulation through coregulation, or the processes by which they provide external support or scaffolding as children navigate their emotional experiences. The vast majority of research to date has examined coregulation in the context of caregiver–child dyads. In this paper, we consider emotion regulation and coregulation as family-level processes that unfold within and across multiple family subsystems and explore how triadic and whole family interactions may contribute to the development of children’s emotion regulation skills. Furthermore, we will examine the implications of a family-centered perspective on emotion regulation for prevention of and intervention for childhood emotional and behavioral disorders. Because emotion regulation skills undergo such dramatic maturation during children’s first several years of life, much of our focus will be on coregulation within and across the family system during early childhood; however, as many prevention and intervention approaches are geared toward school-aged children and adolescents, we will also devote some attention to later developmental periods.

## Introduction

The ability to regulate one’s emotions is foundational for healthy development and functioning in a multitude of domains (Blair et al., 2015; Di Maggio et al., 2016; Graziano et al., 2007; Panlilio et al., 2018). Conversely, impaired emotional regulation has been identified as a transdiagnostic risk factor for many mental health disorders (Aldao, 2016; Eisenberg et al., 2010; Fernandez et al., 2016; Heleniak et al., 2016; Raver et al., 2017). As a result, emotion regulation is viewed as a critically important developmental task that should be promoted for all children and especially for children facing risk.

Because the ability to self*-*regulate emotions is linked to so many other aspects of children’s functioning, it is not surprising child and family researchers have focused considerable attention on coregulation, or the processes by which caregivers^1^ provide external regulation or scaffolding for a child to facilitate the development of emotion regulation over the first several years of life. To date, much of this research has explored coregulation as an exclusively dyadic process (most often between a mother and child) that occurs in a vacuum. However, coregulation is shaped by and shapes the larger family system and likely unfolds in distinct ways within and across different family subsystems**.** Caregivers must attend to and respond to the emotional needs of multiple family members, and children must monitor, process, and respond to multiple sources of input into their emotional lives.

In this paper, we explore emotion regulation and coregulation as family-level phenomena, including how such phenomena play out within and across multiple family subsystems (including caregiver–child, coparents, and sibling relationships). It will also highlight findings on the role of triadic and whole family interactions as important contexts for the emergence of children’s emotion regulation skills. Furthermore, we will examine the implications of a family-centered perspective on emotion regulation for prevention of and intervention for childhood emotional and behavioral disorders. Most extant intervention and prevention programs view parent–child interactions as the primary or sole target of intervention, with relatively little attention paid to other coregulatory processes in the family, including the role that coparent, sibling, or whole family coregulation may play in supporting the development of children’s self-regulatory capacities. Because emotion regulation skills undergo such dramatic maturation during children’s first several years of life, much of our focus will be on early childhood research; however, as many prevention and intervention approaches are geared toward school-aged children and adolescents, we will also devote some attention to later developmental periods. Integrating basic family science with emerging empirical support for innovative interventions that address emotion regulation at the family level, we will close by making recommendations for future work in this area.

### Conceptualizations of Emotion Regulation

Numerous definitions have been proffered to capture the contours of “emotion regulation;” these definitions generally converge around the processes or competencies that involve awareness, evaluation, maintenance, and/or modulation of emotional states to accomplish one's goals (see Calkins, 2010; Thompson, 1994). Emotion regulation may be conscious and deliberate, or unconscious and automatic; self-managed or externally supported (e.g., caregiver soothing a crying infant); and may occur in the context of both positive and negative emotions. Given the complex set of processes that emotion regulation encapsulates (including physiological, cognitive, and social), it is widely viewed as an ongoing developmental task, changing significantly from birth, across childhood and adolescence, and even into adulthood.

Early childhood is a particularly important time in the development of children’s emotion regulation (Calkins, 2010; Sroufe, 1996), as young children shift from being highly dependent upon external regulation from caregivers toward a capacity to exercise increasingly deliberate control over their emotional lives (Grabell et al., 2019; Morelen et al., 2016). As early as infancy, there is evidence for the use of different emotion regulation strategies, including “self-regulatory” strategies such as looking away or self-comforting, and what have been described as “hetero-regulatory” strategies, such as protesting or smiling, that aim to enlist the support of an adult caregiver (Riva Crugnola et al., 2011). In early childhood, the development of increasingly complex emotion regulation skills aligns with the growth in young children’s cognitive and language skills, and includes learning to identify emotions, connect emotions to experiences, and respond adaptively to one’s emotional experiences, including modulating the expression of emotions (e.g., Shewark & Blandon, 2015).

In later developmental periods, children and adolescents become increasingly capable of navigating emotional experiences with greater autonomy, often by necessity, as situations that can evoke strong emotions (e.g., frustrations at school, interpersonal conflicts with peers) may occur when the child or adolescent is not with their caregiver(s). As the child or adolescent moves toward functioning with increased independence, caregivers’ coregulation efforts are likely required to shift away from direct in-the-moment support (Brand & Klimes-Dougan, 2010). Caregivers may instead assume a more behind the scenes role wherein their scaffolding remains important but may come in the form of anticipatory coaching, helping their child or teen prepare to handle a potentially dysregulating situation (e.g., talking through how to address a disagreement with a friend), or “debriefing” after an emotionally distressing event. Particularly during adolescence, caregivers are faced with the challenge of simultaneously attending to their child’s need for greater autonomy while also feeling pulled to intervene by the heightened emotionality that can characterize this developmental period (Van Lissa et al., 2019). The changing landscape of children’s emotional needs from infancy through adolescence highlights the importance of establishing a relationship early on in which the child learns that they can depend on their caregivers for flexible and attuned coregulation that is aligned with their regulatory capacities at each stage of development.

### Emotion Regulation in Relational Context

Much of the research examining the development of children’s emotion regulation is rooted in attachment theory (Bowlby, 1969, 1973). A significant body of research across a diverse range of sociocultural groups has documented the linkages between secure attachment relationships–that is, relationships in which the infant trusts that the caregiver will respond consistently and sensitively to their emotional and physical needs–and the emergence of effective emotion regulation skills in young children (Cassidy, 1994; Gilliom et al., 2002; Panfile & Laible, 2012; Posada et al., 2016; Qu et al., 2016; Riva Crugnola et al., 2011; Waters et al., 2010). In the context of attachment relationships, caregivers are seen as playing a critical role in their children’s emotion development when they function as both a “secure base” and as a “safe haven.” When functioning as a secure base, the caregiver’s primary focus is on supporting the child’s exploration of their social and physical environments. The caregiver may share in and encourage the child’s excitement, or help the child navigate uncertainty or anticipatory anxiety by providing reassurance regarding the child’s abilities (“I know you can do it!”) or the caregiver’s availability (“It’s okay, I’m here if you need help”). When functioning as a safe haven, the caregiver’s primary focus is on comforting or reassuring a child when they become dysregulated. These early efforts to soothe a distressed infant begin building the child’s expectations of what kind of responses they might expect when expressing certain emotions (Thompson, 1994). The caregiver may help the child tolerate and manage feelings such as anger, fear, or sadness, ultimately guiding them back to a more regulated state in which they feel ready to explore again.

Throughout childhood, caregivers continue to play a key role in scaffolding the young child’s emotional experiences, guiding them toward the use of increasingly sophisticated and intentional regulatory strategies (Crugnola et al., 2013; Eisenberg et al., 1998; Gottman et al., 1996; Grabell et al., 2019; Lincoln et al., 2017; Morris et al., 2007; Thompson, 2014). Several studies have demonstrated that caregiver responses to children’s expressions of emotions shape how children learn to communicate about and manage their emotions (Lindsey, 2020; Perry et al., 2020; Shewark & Blandon, 2015). When parents respond supportively to children’s displays of emotions (e.g., validating, encouraging expression, soothing when needed), children are more comfortable experiencing and expressing a range of emotions–both positive and negative–and increasingly secure that such expressions do not compromise the relationship or run the risk of eliciting punitive or withdrawing responses from key caregivers. Furthermore, caregivers’ supportive responses to children’s negative emotions may help children remain organized in the face of elevated physiological arousal (Perry et al., 2020), and thus more capable of accessing their own regulatory strategies, and more receptive to their caregivers’ efforts to provide coregulation. The variability in caregivers’ responses to children’s emotion expressions in different types of situations also provides important social and contextual information. For example, caregivers’ punitive or minimizing responses (i.e., suppression responses) to children’s negative emotions are often linked to poorer child emotion regulation (including reported, observed, and psychophysiological indicators; Morris et al., 2017). However, for Black children, caregivers’ suppression responses to negative emotions, when paired with racial socialization strategies, are associated with an indicator of more adaptive physiological regulation, fewer externalizing problems at age 5 (Dunbar et al., 2021) and fewer depressive symptoms in adulthood (Dunbar et al., 2015).

Caregivers may also provide explicit guidance in identifying and understanding emotions by narrating children’s experiences (e.g., “I think you got scared when the dog ran up to you”). When caregivers help children recognize and understand the emotions they are experiencing, children may become better at anticipating situations or events that elicit specific emotional responses and may feel more prepared to manage them. Thompson and Meyer (2014) have emphasized the importance of conversations about emotions, noting that “such conversations are important because they offer young children insight into the underlying, invisible psychological processes associated with emotion, such as how feelings can be evoked by satisfied or frustrated desires, accurate or inaccurate expectations, or memories of past events” (p. 23). Caregivers may also intentionally model or coach children through the use of various emotion regulation strategies, such as refocusing attention or cognitive reappraisal, as a way to tolerate or mitigate negative emotions (Lincoln et al., 2017; Morris et al., 2011).

Caregivers’ own regulatory capacities may also play a significant role in shaping their children’s development of emotion regulation (Are & Shaffer, 2016; Morelen et al., 2016; Tan & Smith, 2019; Yan et al., 2021). Parenthood has been characterized as an experience that elicits intense, complex emotions (Hajal et al., 2019) and calls upon caregivers to help regulate not only their children’s emotions, but their own as well. Caregivers’ modeling of emotion regulation strategies may communicate to their child–for better or for worse–what is normative and expected in their family with respect to how emotions are expressed and managed. In addition, when caregivers are highly dysregulated–whether in response to their child’s distress or to some other stressor–they may have more difficulty accessing the parenting practices that would typically help their child navigate negative emotions. In contrast, well-regulated caregivers who are not being overwhelmed by their own emotions may be better positioned to think flexibly and try different coregulation strategies. Moreover, children of well-regulated caregivers may be better able to avail themselves of the emotion scaffolding provided because their attention is not being diverted to concerns about their caregiver’s current emotional state.

### Sociocultural Context

Although emotions are a universal part of the human experience, norms around their expression are culturally and contextually specific. Thus, while the core relational processes that support the development of emotion regulation occur across cultures and contexts, sociocultural variation exists in how these processes play out within and across family interactions. For example, the major tenets of attachment theory (e.g., secure base, safe haven) and proportions of children with secure (versus insecure) attachment classifications have been replicated across a wide range of cultural, linguistic, and socioeconomic groups, as well as various family compositions. Yet, specific manifestations of attachment relationships may vary across groups. For example, contingent responding—or, a caregivers’ clearly connected and coordinated response to an infants’ signal—is foundational to developing patterns of coregulation and, ultimately, secure attachment relationships. Although contingent responding as a phenomenon within infant-caregiver dyads appears to be universal, the mechanism of responding varies across cultures. For White families of European origin, the primary mode is face-to-face interaction, including caregivers mirroring their infants’ facial expressions (Beebe et al., 2016; Messinger & Fogel, 2007). For families in parts of Africa, Asia, and the Pacific Islands, touch appears primary (Kärtner et al., 2010; Lavelli et al., 2019; van Ijzendoorn & Sagi-Schwartz, 2008). Levels of acculturation and enculturation are also impactful; for example, mothers who had immigrated from West Africa to Italy showed a mixture of face-to-face, cooing vocalizations seen in their native Italian counterparts, as well as vestibular stimulation more typical of Nso mothers living in Cameroon (Lavelli et al., 2019).

Similarly, cultural and contextual norms and expectations, as well as various aspects of the environment in which a family lives (including sociopolitical and economic characteristics), may impact how caregivers respond to their children’s displays of emotions, and the impact of those parental responses on children’s development (Cole & Tan, 2007). For example, the impact of parental suppression responses to children’s negative emotions on children’s overall socioemotional adjustment appears to vary according to race, ethnicity, and socioeconomic and sociopolitical factors. For example, for European American children, parental suppression responses have been linked to poorer emotion regulation (Morris et al., 2017). Yet, for Latin American and Black children, parental suppression of negative emotion expressions is not associated with poorer outcomes (Labella, 2018; Pintar Breen et al., 2018); in fact, it appears to be associated with better physiological regulation and mental health if paired with adaptive racial socialization practices (Dunbar et al., 2015, 2021). Researchers posit that these ethnoracial differences reflect the fact that Latin American and Black parents’ suppression of their children’s negative emotions—when contextualized within the sociopolitical context that Black and Brown children are disproportionately treated punitively in a variety of settings (e.g., in school, by law enforcement)—is adaptive because it prepares these children for bias. Thus, Latin American and Black parents’ suppression of their children’s negative emotions is intertwined with the adaptive racial socialization process of preparation for bias (Dunbar et al., 2017). When considering emotional development, it is critical to consider not only the relational context, but also the larger cultural and sociopolitical context.

## Expanding the Focus to Whole Family Systems

Although such studies have significantly advanced our understanding of the rich relational contexts in which emotion regulation develops in children, the vast majority of such research has remained focused on caregiver–child relationships. And although emotion regulation has been characterized as a dyadic process (Calkins, 2010), we would highlight, in alignment with other researchers (Butler, 2011; Fosco & Grych, 2013; Lunkenheimer et al., 2012), that it is often much more. Fosco and Grych (2013) noted that “the extant literature on children’s emotion regulation reflects a family context dissected into constituent parts” and that “we do not have an adequate understanding of how the family—the earliest and most potent interpersonal context—shapes children’s emotion regulation” (p. 558). Thus, there is great value in the integration of family systems theory– which emphasizes the manner in which these “constituent parts” (whether caregiver subsystems, child subsystems, caregiver–child dyads, etc.) are interrelated, hierarchical, and self-organizing, and how they adjust (and may even thrive; Henry et al., 2015) in response to stress and perturbation (Cox & Paley, 1997; Minuchin, 1985)–into research on the development of emotion regulation.

In this vein, several scholars have proposed theoretical models integrating family systems and attachment theories. Pérez and colleagues (2017) proposed that although attachment was originally conceived in the context of the relationship between infant and caregiver (usually focusing on the mother), children often navigate their affective experiences in “multi-person contexts,” and thus emotional exchanges with caregivers do not occur in a dyadic bubble. Extending the notion of caregiver as secure base, Byng-Hall (1995) proposed the concept of a “secure family base” that “provides a reliable network of attachment relationships in which all family members of whatever age are able to feel sufficiently secure to explore" (p. 46) as they share the expectation that their emotional needs will be met within the family. The notion of a “secure family base” further highlights that coregulation does not occur exclusively within caregiver–child interactions, but also within and among all the various subsystems within a family. As a specific example, some scholars have integrated attachment and systems theories to conceptualize family functioning and reorganization during parental military deployment cycle (including preparation, deployment, and reintegration), which is viewed as a perturbation that reverberates across the family system (Paley et al., 2013; Riggs & Riggs, 2011). Empirical work drawing on these theoretical models indicates the need to consider complex interactions across family subsystems in order to understand the emotional adjustment of military-connected children. For example, one study showed that the association between fathers’ perceived threat during a previous deployment and their preschool-aged children’s social–emotional adjustment was mediated not only by father-reported parent child interaction, but also by maternal reports of family-level emotional responsiveness (Hajal et al., 2020). A longitudinal study showed that school-aged children’s social–emotional adjustment over the course of a parents’ deployment was associated with sibling relationship quality, even beyond the impact of the non-deployed caregiver’s parenting practices (Whiteman et al., 2020).

Furthermore, in addition to the need to consider multiple family dyads, there is a growing body of literature documenting the quality of triadic interactions as a unique predictor of children’s emotional and behavioral functioning, even after accounting for the contribution of individual and/or dyadic family processes (e.g., Hollenstein et al., 2016; Mchale & Rasmussen, 1998; Murphy et al., 2017). In these next sections, we focus on two family subsystems that are critical to children’s emotional development but that are less frequently studied in this area: the co-caregiving dyad and the sibling dyad. Furthermore, we discuss how each of these dyads inter-connects with the caregiver–child dyad in the context of children’s developing emotion regulation.

### The Caregiver System

#### Coregulating as a Coparenting Team

Because emotion regulation often occurs in multi-person contexts, it requires all parties to monitor and navigate multiple streams of emotion-laden interactions that are constantly shifting. Thus, in families with two caregivers (e.g., coparents, grandparent and parent), effective coregulation of a child likely relies not only on each caregiver’s sensitive and contingent responses to the child’s emotional needs, but also on caregivers’ capacity to both coordinate with and support one another in those responses. A study of African American mothers and fathers (75% non-coresidential), for example, found that during triadic interactions with their 3-month-old infants, a substantial portion of the time fathers responded to infants’ bid for engagement with affective and behavioral matching, and mothers supported these father-infant interactions the vast majority of the time (Coates & McHale, 2018).

Importantly, such coordination does not necessarily require that coparents utilize the same coregulation strategies or respond to the same aspects of the child’s emotional experiences. Feldman (2003) found in caregiver–child dyadic interactions that mothers and fathers played important but distinct roles in providing emotion scaffolding for their infants with mothers coordinating socially oriented exchanges of affect, and fathers providing management during bouts of intense positive arousal. Such findings raise the question of whether coparents replicate these different but potentially complementary strategies in triadic interactions. If coparents can work together in coordinated ways, it can model for the child how relationships can be utilized in a healthy way to manage difficulty emotional experiences. Moreover, caregivers’ ability to effectively coregulate the child as a coparenting unit is likely to be reassuring to the child (Byng-Hall, 1995), whereas caregivers’ evident challenges in such coordination are likely to be dysregulating to the child. Effective coregulation may also further consolidate the child’s and the caregivers’ experience of the family as a secure base, shoring up their confidence that the dysregulating experiences that will invariably arise in future can be successfully managed in their family.

On the other hand, coregulating as a coparenting team may be quite challenging when caregivers have different approaches to responding to a dysregulated child, which may impact both their individual and any joint efforts to coregulate the child. For example, if one caregiver has a strong belief that stepping in quickly to respond to their child’s distress will lead the child to be poorly behaved, that may discourage the other parent who wants to respond promptly to the child’s emotional needs. These differences may arise as a function of caregivers’ current regulatory capacities, as well as their beliefs about how emotions should be expressed and managed, or what Gottman et al. (1996) referred to as “meta-emotion philosophy.” Caregivers may also carry patterns of emotion regulation and coregulation into the current family system that have been shaped by their experiences in relationships with intimate partners as well as their respective attachment histories (see Paley & Hajal, 2021). For example, in comparison with mothers with insecure attachment histories, secure mothers are better at recognizing infant emotion (Leyh et al., 2016) and provide more effective coregulation for their infants (Crugnola et al., 2013).

Such differing approaches may give rise to active disagreements over how one parent is responding or not responding to the child’s distress so that the coparents’ conflict then becomes layered onto the child’s initial distress, further dysregulating the entire family. Moreover, children may be faced with contradictory messages within the same interaction regarding what is expected in their family in terms of expressing and managing emotions. Furthermore, in highly dysregulated contexts, the emotional, cognitive, and physiological resources of caregivers and children could become easily overwhelmed by the complexity of attending to, interpreting, and responding effectively to the multitude of emotional exchanges. And as a family grows, the number of subsystems within the family quickly multiplies, and each subsystem may be characterized by its own distinct patterns of emotion interactions (Caldera & Lindsey, 2006; Salman-Engin et al., 2018). Family members’ capacity for self-regulation and coregulation may vary across subsystems depending on who is part of that subsystem (Feldman, 2007). For example, a parent may be able to effectively self-regulate and coregulate during a dyadic interaction with their child, but find it highly dysregulating when the partner and/or another child enter the interaction.

There may be times when children are allowed or actively enlisted to provide coregulation for other family members or subsystems (i.e., child-to-parent or child-to-co-parenting dyad). Byng-Hall (1995) has described instances in families which lack a secure family base where “an individual may turn to an inappropriate member of the family. For example, this may occur when one parent is, for one reason or another, not acting as a secure base for the other parent, who may turn to a child instead. This can undermine the parent's care of the child” (p. 46). Children, even relatively young children, may also be motivated on their own to respond to dysregulated coparent dyads in order to re-establish their sense of emotional security in the family (Davies & Cummings, 1994). A wealth of studies have documented the adverse effects of interparental conflict on children’s emotional well-being (Harold & Sellers, 2018; van Eldik et al., 2020). Thus, whether they are in the role of observer or active participant, children may seek to prevent or reduce such conflict, by monitoring the interactions in the coparenting subsystem, and at times stepping in to provide coregulation, whether by distraction, pleas, admonitions, or other efforts aimed at disrupting negative emotional exchanges between their caregivers. Such occurrences highlight that lapses in coregulation between caregivers do not just play out between coparents but can ripple throughout the family unit, wherein children prematurely assume responsibility for managing the feelings of others in ways that are misaligned with their developmental capacities.

Much of the research on coparenting to date has focused on exploring the ways in which coparenting dynamics–particularly those related to affective exchanges between caregivers–may shape the development of children’s emotion regulation. However, consistent with a family-level perspective, there is a recognition of the mutual influences between the coparenting subsystem and the child. Morris et al. (2007) have noted that in their model, “children’s [emotion regulation] and familial influences are bidirectional processes…, supporting a family systems view where children and families mutually influence one another throughout development” (p. 364). For example, a longitudinal study (Gallegos et al., 2017) of the association between dyadic and triadic predictors of child emotion regulation found that father–child withdrawal at 8 months and coparenting conflict at 24 months were both associated with less adaptive child emotion regulation at 24 months. However, an alternative model in which child emotion regulation predicted coparenting conflict provided an equally good fit for the data.

Such findings underscore that while dysregulation in the coparent subsystem may impact children’s emotional well-being, children who are significantly dysregulated also may have an adverse effect on the relationship between their caregivers. However, the mechanisms by which dysregulation in a child may lead to dysregulation in the coparent subsystem merit more investigation. One possibility is that if coparents feel ineffective in managing their child’s dysregulation, they may displace their frustration and look to blame their partner. It is also possible that dysregulation in a child may illuminate differences between caregivers along a number of dimensions–how emotions should be expressed in the family, how quickly caregivers should respond to their child’s dysregulation, and what coregulation strategies caregivers should use (or not use). Furthermore, such differences may become even more of a flashpoint in the coparenting relationship if they echo disagreements about how emotions are navigated in the caregivers’ intimate relationship. Given how normative it is for children in particular to have periods of dysregulation, there would be considerable value in developing a better understanding of the pathways by which their dysregulation may impact the larger family system.

#### Adult-to-Adult Coregulation

Although much of the work on coregulation has understandably focused on caregiver–child dyads, there is a growing body of research aimed at understanding this process in the relationships that caregivers have with one another. Adults can play a significant role in helping their partners manage their emotions in response to both daily stressors and major challenges (Butler & Randall, 2013; Sbarra & Hazan, 2008). As a coparenting team, caregivers ideally navigate their responses to their child’s dysregulation as a coordinated unit. However, such coordination is likely enhanced by how the caregivers also respond directly to one another before, during, and after these periods of child dysregulation. Indeed, adult-to-adult coregulation has been increasingly recognized as a critical piece of parents’ ability to self-regulate and provide coregulation to their children. Importantly, when adults transition into parenthood (for those with partners), the relational unit expands from dyad to triad (or beyond), which entails a major reorganization of family dynamics wherein two adults are no longer just romantic partners, but now coparents as well. An adult who previously needed to focus only on their own and their partner’s emotional needs now has to redistribute (and potentially reprioritize) their attention and their responses to both their child’s *and* their partner’s emotional needs. Responding to children’s emotions can be exhausting, overwhelming, and at times, highly dysregulating for caregivers (Hajal & Paley, 2020), and it is likely such interactions may activate caregivers’ own needs for their partners to act as a secure base. That is, it may be easier for caregivers to stay regulated themselves if they know they can share the emotional weight of supporting a highly distressed child.

A number of studies have documented the linkages between coparenting interactions marked by warmth and cooperation and children’s regulatory competencies (Feinberg et al., 2009). Similarly, studies have documented the contributions that triadic interactions make to the prediction of children’s emotional well-being. A recent study by Perez et al. (2017) supports this notion, with reported findings of an association between positive triadic (i.e., mother-father–child) interactions and children’s secure attachment representations assessed in preschool. León and Olhaberry (2020) also found evidence for the contribution of triadic interactions to children’s outcomes, with the quality of triadic interactions acting as a mediator between maternal reflective functioning and social–emotional competency among children aged 12–36 months. When caregivers are able to establish the coparenting subsystem as its own harmonious and mutually supportive unit, it may build both the caregivers and the child’s confidence that there will be a stable and secure family base to help them weather the emotional storms of childhood.

Another important component of adult-to-adult coregulation entails how caregivers process parenting-related affective experiences with one another in the absence of the child. For example, when a partner responds empathically to a caregiver who has had a frustrating experience with their child, it may help normalize the caregiver’s response while also allowing them to “let go” of residual negative emotions. The partner may even gently prompt the caregiver to consider the child’s perspective, and to reappraise where the child is developmentally to normalize what might be experienced as hurtful behavior from the child. A caregiver who is feeling rejected by the child may be soothed by their partner’s reassurances of how important they are to the child and how typical it is for 4-year-olds to say “I hate you” when they are angry, perhaps prompting the caregiver to respond in a more regulated fashion during their next interaction with their child. Thus, ongoing coregulation within one subsystem may shape coregulation in another subsystem not only within a specific interaction, but also across interactions over time.

Through these interactions, caregivers may help one another modulate the emotional experiences of parenting, amplifying positive experiences that may help serve as an important counterweight to more difficult moments, and mitigating negative experiences by validating and normalizing the challenges of parenting. Adult-to-adult coregulation skills can play a key role in shaping both caregiver–child and whole family relationships. Partners can fortify one another to recover from distressing interactions and to prepare for the next parenting challenge that will inevitably arise. Effective adult-to-adult coregulation may also motivate a parent to make repairs and re-establish harmonious interactions with their child. Similar to the safe haven and secure base functions that adults serve in parent–child relationships, coparents may serve in similar roles for one another. In providing reassurance and comfort to a frustrated or distressed partner that allows them to re-engage in a more regulated way with their child, coparents may be allowing one another to engage in a process analogous to the “smooth transitions” (Ainsworth, personal communication, as cited by Grossmann & Grossmann, 2020) “between the activated attachment system and the exploratory system [that] are in fact the central marker of a secure attachment” (p. 3). In essence, adult-to-adult coregulation may act in a parallel fashion to adult-to-child coregulation where all family members are able to move back and forth between reassurance-seeking and exploration because of a secure family base.

### Child-to-Child Coregulation

Sibling relationships have the potential to provide young children with some of their earliest experiences of intense positive (e.g., joy, excitement) and negative (e.g., frustration, anger, jealousy) emotions (Kramer, 2014; Lindsey, 2020). Furthermore, there is evidence that the quality of sibling relationships is a unique predictor of children’s psychosocial outcomes, beyond the contributions of caregiver–child relationships (McHale, Updegraff, et al., 2012; McHale, Waller, et al., 2012; Modry-Mandell et al., 2007; Stormshak et al., 2009; Whiteman et al., 2020). Sibling interactions may provide opportunities for children to observe their siblings model adaptive emotion regulation skills or to benefit from siblings who provide them with coregulation during moments of distress (Kramer, 2014). For example, a study of older children’s reactions to their young siblings’ emotions demonstrated that older siblings’ positive response to emotions predicted their young siblings’ emotion knowledge (Sawyer et al., 2002).

The emotional landscape of sibling relationships is not exclusively shaped by the siblings themselves, as it may also be influenced by other family members and subsystems. In an observational study of triadic interactions among mothers and their children, Kojima (2000), for example, found that when mothers drew older siblings’ attention to their younger siblings’ emotion states, older siblings exhibited more positive behavior toward their young siblings. In another study of toddler and preschool-aged children, Yaremych and Volling, (2020) reported that fathers’, but not mothers’, supportive and nonsupportive emotion socialization practices were associated with negative emotions in the sibling relationships. At times, the emotional intensity of sibling interactions may require caregivers to provide coregulation to two or more highly dysregulated children, each of whom may have diametrically opposed perspectives on who is responsible for their distress and what remedy might resolve their distress. Thus, caregivers are often tasked with the challenge of meeting (often simultaneously) the distinct emotional needs of each child during sibling interactions. Moreover, how they meet those needs may have implications for subsequent sibling and caregiver–child interactions, particularly if children feel their caregiver is more responsive to their sibling’s needs or interests than to their own (Kramer, 2014). A number of studies have found that parental differential treatment–based both on siblings’ perceptions and on parent report–is associated with higher levels of dysregulation in sibling relationships (Meunier et al., 2012; Richmond et al., 2005; Volling & Elins, 1998).

Efforts to support the sibling relationship may become even more complicated if another caregiver is involved, particularly if caregivers have different approaches to intervening in sibling conflicts, potentially leading to formation of family alliances that cause divisions within the coparent and sibling subsystems (i.e., each caregiver is experienced as more responsive to one sibling versus the other). On the other hand, sibling relationships may also moderate the impact of other family relationships on children’s well-being. Sibling relationships in which children provide mutual emotional support to one another may mitigate or buffer the negative effects of dysregulation in other family relationships (e.g., interparental conflict; Davies et al., 2019; Milevsky & Levitt, 2005; Thompson, 2014). Such findings support the notion that how emotions are navigated in sibling interactions is influenced by and influences other family subsystems. However, there is a need for more longitudinal research that explores the likely reciprocal influences that these family subsystems have on the patterns of emotion regulation and coregulation that are established among different family members.

### Triadic and Whole Family Coregulation

As noted earlier, triadic and whole family interactions appear to make unique contributions as predictors of children’s emotional competence even after accounting for the quality of dyadic caregiver–child interactions (Mchale & Rasmussen, 1998; Murphy et al., 2017). A caregiver may interact differently with their child as a function of the involvement or even mere presence of their coparent. For example, both mothers and fathers were found to interact more sensitively with their infant in the presence of their coparent as compared to when they interacted with their child in the absence of their coparent, but only when the family was characterized by a “high coordination” alliance (Udry-Jørgensen et al., 2016). Thus, in families characterized by qualities such as family warmth, validation of the child’s emotional experiences, and the inclusion of partners, the presence of the coparent may serve a regulating function that allows the caregiver to be more attuned to the child’s emotional needs. Triadic and whole family interactions also allow for the observation of processes that could not otherwise be discerned in the context of dyadic exchanges between caregiver and child, such as when one family member is excluded by two other family members (Hollenstein et al., 2016).

In line with the increased interest in moving beyond the dyad, a small but growing number of studies have focused on how emotional exchanges are navigated in the context of triadic and whole family interactions (Fivaz-Depeursinge et al., 2012; Hollenstein et al., 2016). For example, in studies utilizing the Lausanne Trilogue Play (LTP; Corboz-Warnery et al., 1993), a staged paradigm used to assess how a family of three navigates interactions when all members are present, infants as young as three and four months demonstrated “triangular capacities” or the ability to flexibly shift their gaze and affect between two caregivers (McHale et al., 2018). In another study utilizing the LTP paradigm with nine-month-old infants, the triadic interactions of low coordination families were compared with those of high coordination families (Fivaz-Depeursinge et al., 2012). The infants in the low coordination families displayed less positive affect and attempted fewer positive triangular bids or efforts to engage and interact with both parents at the same time, highlighting that these infants will likely have fewer opportunities to develop the skills to navigate triangular interactions, an importance competency for emotion regulation. Other research has focused explicitly on how emotions are coregulated at the family level and the use of specific emotional socialization practices. In a study of school-aged children, greater flexibility in family members’ expressions of affect during whole family interactions was linked to higher levels of emotion regulation in children (Lunkenheimer et al., 2012). Moreover, this study found that family interactions in which emotions were dismissed during positive conversations were associated with lower emotion regulation skills, whereas elaborating on discussions of emotions during difficult conversations was linked to higher emotion regulation skills. Taken together, these investigations add to a gradually emerging body of work highlighting that the processes that support or hinder the development of emotion regulation occur not only within the caregiver–child dyad, but also unfold within and across subsystems that impact and/or directly involve multiple family members.

## Implications for Prevention and Intervention

Given the clear role of emotion regulation as a foundational process that, when developing optimally, contributes to healthy development, but when gone awry, introduces risk for a variety of psychological disorders, it is a critical target for many psychosocial prevention and intervention approaches. For young children, whose ongoing development in a variety of domains makes their general emotion knowledge and skills more limited than older adolescents and adults, prevention and intervention programs often start with the building of basic emotion knowledge, such as acquiring and practicing an emotion vocabulary and developing an understanding of nonverbal emotion cues (e.g., facial expressions). Given that emotion regulation capacities develop over the course of childhood and adolescence, aspects of caregiver–child coregulation are incorporated into many of the evidence-based prevention and intervention programs for youth.

### Prevention and Early Intervention

Given the centrality of the caregiver–child relationship for the development of emotion regulation in early childhood, virtually all evidence-based prevention and early intervention programs for infants, toddlers, and preschool-aged children heavily incorporate the primary caregiver. Because young children are reliant on adults for external regulation and scaffolding of self-regulation, it is logical for caregivers serve as the primary vehicle for therapeutic change. Furthermore, the most common risk factors for psychological maladjustment in early childhood are related to vulnerabilities in the caregiver–child relationship, such as caregiver depression or children’s early separation from attachment figures due to welfare system involvement, parental loss, or other challenges. As a result, many early childhood interventions target caregivers’ own emotion regulation, particularly in the context of children’s distress. For example, several evidence-based programs, including individual family dyadic interventions, such as Attachment Biobehavioral Catch-Up (Dozier & Bernard, 2017) and Child–Parent Psychotherapy (Lieberman et al., 2006) and caregiver group-based interventions, such as Circle of Security (Hoffman et al., 2006) and Tuning Into Toddlers (Lauw et al., 2014), share a primary goal of promoting healthy child development by increasing caregivers’ sensitive parenting and adaptive emotion socialization strategies, and view caregivers’ own emotion regulation as a precursor to this capacity. Thus, providers work with caregivers on identifying their own emotional responses to challenging parenting situations (including exploring how certain behaviors or signals from children may be evocative of other events in the caregivers’ personal history) and teach adaptive emotion regulation strategies. Although most of these interventions do not systematically engage family systems and subsystems beyond the parent–child dyad (although some, such as Attachment Behavioral Catch-Up, engage coparenting figures when available), the degree to which caregivers’ own emotions are targeted often elicits discussion and problem-solving around the other family relationships that are intertwined with the caregiver–child dyad (e.g., romantic partner, coparent, caregiver’s family of origin relationships).

### Treatment for Child and Adolescent Psychological Disorders

Beyond prevention and early intervention, many of the most well-supported approaches for treating common disorders such as anxiety, mood disorders, behavioral problems, and traumatic stress in older children and adolescents (as well as adults) include psychoeducation, training, and practice in multiple aspects of the emotion regulation process including awareness, monitoring, evaluation of an experienced emotion in relation to one’s goals, and a variety of strategies to modulate emotions to best serve those goals. Integration of emotional coregulation as an intervention target can take a variety of forms, including parent-only psychoeducation and skill-building, as well as in vivo guidance in emotion coaching within dyadic interventions (e.g., conduct problems; Eyberg & Bussing, 2011; depression; Luby et al., 2018) or during conjoint sessions occurring within a predominantly individual child treatment (e.g., traumatic stress, Cohen & Mannarino, 2015; anxiety, Podell et al., 2010). The specific manner in which emotional coregulation is integrated may differ depending on the child’s developmental stage and presenting challenge, but ultimately these approaches all recognize that in order for children to make and maintain gains in emotion regulation processes that are associated with their symptom presentations (e.g., anxiety, traumatic stress, depression), they need caregiver support. Increasingly, interventions are also recognizing the impact of caregiver well-being on children’s ability to benefit from treatment by adding treatment module or activities focused on caregiver’s own emotion regulation (e.g., trauma, Cohen et al., 2017; externalizing behavior problems, Sanders & Mazzucchelli, 2013).

### Enhancing Emotion Regulation Across the Family System

Although caregiver–child coregulation is undoubtedly important, it is notable that other family subsystems, not to mention the entire family unit, are not systematically targeted by most youth prevention and intervention programs. This is at odds with the research (reviewed above) indicating the importance of emotion regulation within other family dyads (e.g., co-caregivers, siblings), and larger family systems (i.e., triads and beyond) on children’s emotional well-being. Whole family approaches *do* have a long history in psychosocial prevention and intervention; family therapy approaches (e.g., structural family therapy, strategic family therapy) have long focused on the multiple streams of interaction among and within the multiple family subsystems that make up whole family units. Yet, the focal target of treatment of most traditional family approaches is not emotion regulation specifically, but rather other family structures (e.g., hierarchies, boundaries) or processes (e.g., storytelling, problem-solving; (Tadros, 2019).

This section will discuss ways in which we might bridge across these previously parallel paths of intervention by explicitly targeting emotional processes at multiple levels of a family system beyond the caregiver–child dyad (including the coparent system, the sibling relationship, triads and beyond) via psychoeducation, skill-building, and in vivo family emotion regulation practice. Psychoeducation, which is a core component of most prevention and intervention programs, serves to help individuals understand the nature of their presenting difficulty. Frequently, newly learned information is then applied within the context of skill-building activities that help families attain their treatment goals, and often these skills are practiced in sessions. We include a discussion of some of the innovative work that has already begun to make progress toward this goal.

#### Coparent Subsystem

Psychoeducation and skill-building on family emotion regulation may be done with any configuration of family members, and does not necessarily require all members to be present in order to have an impact on the entire system. For example, enhancing emotional coregulation among caregivers can have a significant impact on child well-being, as improving relationships between caregivers may lead to more coordinated coparenting, modeling of healthy coregulation, and a more positive family emotional climate.

In general, caregivers may benefit from a more explicit understanding of how emotion dysregulation can reverberate throughout the entire family system, including how their expression and management of their own emotions can impact other family members (i.e., children, other caregivers in the family). Psychoeducation regarding emotional coregulation as a family-level process may be especially important in families with young children, as it is not uncommon for caregivers of infants and toddlers to underestimate how attuned young children are to their environments. Infants as young as 9 months old spend more time looking at adults expressing anger than those expressing positive or no (i.e., neutral) emotion (Moore, 2009; Repacholi & Meltzoff, 2007). Yet, in our clinical experience we have found that caregivers have a tendency to dismiss the impact of very early life events on their child, saying “They were just a baby, they didn’t know what was going on.” Even in infancy, exposure to ongoing parental conflict can have generalized and long term effects: 6-month-olds exposed to a high level of interparental conflict or domestic violence (respectively) showed poorer physiological regulation during a mother-infant interaction (Moore, 2010), and heightened sensitivity in response to anger expressed by an unfamiliar adult (DeJonghe et al., 2005). Sensitizing caregivers to how attuned even very young children are to their interpersonal environments may encourage them to view the establishment of a healthy emotional climate in the family as foundational to their child’s development. There are a few types of preventive interventions that engage the coparenting system without the child present, but with the ultimate goal of promoting child development. These programs are generally positioned around family events that are likely to elicit stress and conflict, such as the transition to parenthood and parental separation (e.g., Feinberg & Kan, 2008; McHale, Updegraff, et al., 2012; McHale, Waller, et al., 2012), but, surprisingly, they generally do not explicitly focus on how caregivers coregulate *one another’s emotions.*

Caring for children is an emotional and often stressful endeavor (Bradley et al., 2013; Hajal et al., 2019), and families in which there are multiple caregivers (whether a parent, a non-parental relative, or non-relative caregiver) may benefit from being able to lean on the support of their co-caregiver when needed. This is important for all families, but especially families in which a caregiver is ill. As one example, it is well established that parental depression has a negative impact on children’s socioemotional adjustment; the relation between parental depression and children’s adjustment appears to operate through a variety of factors, including the depressed caregivers’ parenting practices, lack of engagement, and displays of negative emotion (Goodman et al., 2011, 2020; Lovejoy et al., 2000). However, research suggests that sensitive caregiving from a non-depressed caregiver can buffer the negative impact of maternal depression on overall family functioning (Vakrat et al., 2018a) and child socioemotional adjustment (Vakrat et al., 2018b), even in families where there are multiple risk factors (e.g., teen mothers who are depressed; (Lewin et al., 2015). Furthermore, longitudinal research suggests that the buffering effect is long-lasting, including from perinatal maternal depression to 12-month child development (Goodman et al., 2014), and maternal depression during infancy to kindergartner internalizing symptoms (Mezulis et al., 2004). Observational research with infants suggests that the buffering effect of father involvement operates not only through warm or sensitive fathering, but also through its association with increased family cohesion during triadic interactions with the infant and reduced maternal distress (Feldman, 2007). Thus, families in which a caregiver is depressed may benefit not only from individual treatment for the depressed caregiver, but also coparent or family-level regulation that aims to shore up the capacities of the non-depressed caregiver, whether as a support to their partner, their children, or both. Partner-assisted therapies for perinatal depression (in which the depressed caregiver’s partner is involved in at least one session) have been developed, and initial studies suggest promise for this approach, although there are not yet enough data to test whether adding a patient’s partner adds incrementally to established individual therapy approaches (Sockol, 2018). Future clinical research in this area should assess multiple aspects of family functioning, including perceived quality of the coparenting relationship and child adjustment.

Basic family science research suggests several other areas that could be fruitful for intervention developers to consider integrating into their approaches. Many dyadic interventions focus on reciprocal interactions between a young child and a single caregiver (e.g., Dozier et al., 2002; Lieberman et al., 2000; Luby et al., 2018). Yet, there is evidence (McHale & Fivaz-Depeursinge, 1999; McHale et al., 2008) for the emergence of “triangular capacities” in infants as young as 3 months wherein they are able to competently coordinate their attention and the exchange of affect in the context of triadic interactions (i.e., infant interacting with two caregivers). A next step for intervention could be to integrate provider coaching of caregivers during whole family interactions in addition to dyadic interactions.

Additionally, many behavioral parent training approaches involve creating a “parenting plan,” or, advance planning of adaptive strategies that caregivers can use in response to children’s behaviors. Although originally stemming from a heavily behavioral perspective, some contemporary programs integrate discussion of parents’ and children’s emotions into the creation of parenting plans. One potential area for future work is for intervention approaches to create a similar plan for coregulation during coparenting. This might include advance discussion about how best to coordinate efforts to provide coregulation to a distressed child and ensuring they are not working at cross purposes. For example, if one caregiver is attempting to distract a dysregulated child, it would be important for the other caregiver to follow their lead rather than trying to re-focus the child on a discussion of why they are upset. A caregiver might also reinforce the existence of a secure family base by overtly narrating and praising the other caregiver’s efforts to provide coregulation for the child (“I bet that hug from Grandma is helping you feel better!”). Caregivers might also work together to navigate emotionally charged family interactions by allowing one another to “tap out,” stepping in to soothe a distressed child when the other caregiver needs a break. This kind of coordination can be difficult to execute when family interactions are highly dysregulated, so it might be beneficial for caregivers to communicate and plan in calmer moments about how they might work together as a team in those more dysregulated moments.

The value of intentional discussions about how to approach coregulation as coparents also extends to communication that can happen after emotionally charged family interactions. Emotion regulation in family systems is a dynamic process (Butler, 2011; Calkins, 2010), in which dysregulated interactions can continue to echo through the family even long after specific interactions have ended. By periodically debriefing outside of the heat of the moment, coparents can reflect on both their own and other family members’ experiences of the interactions. Taking time to process and deconstruct emotionally challenging interactions with their child may strengthen the coparenting dyad by allowing them to clarify miscommunications, discuss differing perspectives on how to manage a highly distressed child, and allow them to plan for any different strategies they might want to try in the future, all of which may lessen the negative residue of such moments. Moreover, as children mature, they can be included (when appropriate) in these post-dysregulation discussions to further reinforce the norm that emotional experiences can be openly discussed and navigated as a family.

#### Sibling Subsystem

Perhaps even more than the coparent subsystem, the sibling relationship has been relatively neglected in terms of emotion-focused prevention and intervention. Given the impact that siblings have on one another’s emotion socialization (Kramer, 2014) and development of psychopathology (Buist et al., 2013; Feinberg et al., 2012; Kramer & Conger, 2009; Whiteman et al., 2020), this is a significant gap. Furthermore, the quality of the sibling relationship has an impact on the larger family system; for example, one study showed that sibling agonism had an impact on parent emotional reactivity and regulation (Ravindran et al., 2015). In some family constellations, such as when children are in foster care (Kothari et al., 2017), or experience another type of caregiver separation (e.g., military deployment; Whiteman et al., 2020), the sibling relationship might be the most consistent close family relationship, at least for a period of time. Thus, it may be particularly important to address enhancement of sibling relationships for children who face adversity.

A few sibling relationship interventions exist, all of which address emotions to some extent (Feinberg et al., 2013; Kothari et al., 2017; Updegraff et al., 2016). For one program, The More Fun with Sisters & Brothers Program (MFWSB; Kennedy & Kramer, 2008), promoting family-level emotion regulation is central to the theory of change. MFWSB is based on the idea that emotion socialization and learning occurs partly via sibling relationships (Kramer, 2014). It posits that enhancing sibling *prosocial* behaviors is just as important, if not more so, than reducing sibling conflict and rivalry (Kramer, 2010), and views building emotion regulation capacities as a key skill that can enhance sibling relationships (Kennedy & Kramer, 2008; Kramer, 2010). An individual family intervention, all family members receive psychoeducation in seven key social–emotional skills shown by research to promote positive sibling relationships (Kramer & Gottman, 1992; Kramer & Kowal, 2005), which include identifying emotions, perspective taking, regulating intense emotions, and managing conflict. Most sessions are dyadic sibling sessions that involve in vivo practice, with provider coaching, of the sibling skills; caregivers are heavily involved in “transfer of training” activities such that they can guide the generalization of these skills to the home setting. A randomized controlled trial showed that MFWSB increased sibling prosocial behavior and decreased sibling conflict. In line with the view that the quality of the sibling relationships reverberates throughout the family system, MFWSB also had a significant positive impact on parents’ emotion dysregulation (Kennedy & Kramer, 2008).

There are several challenges in building sibling interventions, which may partly explain the relatively scant attention paid to this family subsystem from an intervention perspective. First, unlike other family subsystems, in which at least one partner is an adult, the sibling relationship involves at least two children, whose developmental stages might vary widely. Current sibling interventions provide at least some age-related boundaries; for example, MFWSB is for families in which both siblings are between 4 and 8 years (Kennedy & Kramer, 2008), the multi-family group-based Siblings Are Special program (Feinberg et al., 2013) is for 5^th^ graders and their younger siblings, and the individual family-based SIBS-Foster Care program (Kothari et al., 2017) requires that the older sibling be between the ages of 11 and 15 years. These age parameters make sense given the wide variety of developmental capacities in the skill areas that these interventions focus on (e.g., perspective taking, social skills, emotion regulation) and the activities that siblings might engage in together (e.g., play for young children, versus a conversation for adolescents). However, there remains a need for programs that address other sibling constellations, not only in age ranges but also in number of siblings, biological relatedness, etc. Future intervention development work might also explore adding sibling relationship psychoeducation and skill-building components on to whole family interventions; some examples of this are described below.

### Whole Family System

Depending on the developmental level of the child, psychoeducation, skill-building, and in vivo practice of family emotion regulation may be implemented at the whole family level. Due to differing capacities of children and adults to learn new information, it may be necessary to initially teach information in separate caregiver and child sessions, before reviewing and practicing skills as a family unit. Or, material may be taught to all family members at once, with providers deferring to the family member at the earliest developmental stage, possibly enlisting older children and caregivers to support the younger child’s learning. Although there has been more basic family science research conducted on coparenting and sibling dyads than triadic-plus family interactions, from the clinical science side, interventions that target the whole family system appear to be more common. Many (although not all) of the family-level interventions that address coregulation are fairly recent (i.e., within the last two decades) adaptations of individual child therapies. These approaches go beyond periodically bringing parents in for collateral sessions (e.g., to report on the child’s behavior at home, or to hear about what their child is learning in therapy) by systematically and actively integrating multiple family members into sessions. Perhaps due to that particular evolution, there has been more empirical investigation in the whole family interventions literature (as compared to coparenting or sibling interventions) of the incremental, added value of considering the whole family in treatment, above and beyond individual child treatment.

CBT is well established for the treatment of anxiety disorders in children and adolescents, with both individual child CBT and individual family-based CBT shown to be efficacious (Goger & Weersing, 2021; Higa-McMillan et al., 2016). Individual family-based CBT (FCBT) was developed with the recognition that a high proportion of children with anxiety disorders also have parents with anxiety, that children’s anxiety can be distressing for parents, and that caregiver behaviors (e.g., modeling of anxious behaviors and maladaptive coping strategies, parental accommodation of children’s avoidance, etc.) contribute to and maintain child anxiety (Goger & Weersing, 2021). Although the degree of caregiver involvement and specific family emotion regulation topics covered vary by specific model, FCBT for anxiety generally includes caregivers in at least some sessions in which they receive psychoeducation on the impact of their own emotional responses to their children’s anxiety and their behaviors (e.g., accommodation, overprotection, modeling) that may be maintaining it, and skills training to enhance their own emotion regulation, parenting, and emotion socialization behaviors (Wood et al., 2009). A substantial number of studies have been conducted on FCBT, and interestingly, evidence for the incremental benefit of FCBT over individual child CBT has been mixed, with several meta-analytic studies showing no benefit (Goger & Weersing, 2021; Peris et al., 2021). It may be that FCBT is incrementally beneficial for specific populations (e.g., families in which parents have an anxiety disorder; Kendall et al., 2008), or that for FCBT to be uniquely helpful, it must include certain parenting components (Manassis et al., 2014). Peris and colleagues noted that the vast majority of FCBT studies include only child diagnosis and/or symptoms as an outcome measure, and surprisingly few included pre- and post-intervention assessment of the targeted family process (Peris et al., 2021). Thus, it is possible that lack of findings for FCBT’s incremental benefit is due not to family processes *not* being an important part of treatment for childhood anxiety, but rather, to current treatment approaches’ lack of efficacy in improving those family processes. Notably, FCBT for child anxiety typically focuses on parents’ individual emotion and emotion regulation and the parent–child dyad, without consistent, particular attention paid to the coparent relationship, sibling relationships, or whole family interactions. This represents a gap in this work, given evidence that partner conflict is a maintaining factor for parental anxiety (Stuart Parrigon & Kerns, 2016) as well as the impact of sibling relationships on emotion socialization (Kramer, 2014) and the development of psychopathology (Feinberg et al., 2012). Thus, in addition to including measurement of the family processes identified as treatment targets (Peris et al., 2021), a next step for FCBT for childhood anxiety might be to incorporate intervention components for other family subsystems.

Another example of an individual child treatment that has been adapted to engage the entire family is Family-Focused Treatment for Childhood Depression (FFT-CD), which has been tested with children aged 7–14 years as an individual family intervention. FFT-CD is unique in its emphasis on family relationships as the primary mechanism to improve depression symptoms, and works across the associations among depressed mood, emotions, and family interactions (Tompson et al., 2020). For example, FFT-CD providers work with family members to fill out an adapted version of the commonly used “spiral” for family interactions with family interactions in mind. These “spirals” are generally used in individual CBT for depression to delineate the way thoughts, feelings, and behaviors influence one another to lead to depressed mood (e.g., feeling sad → withdraws from others → thinks “I’m all alone” → feels more sad, depressed). In FFT-CD, however, the emphasis is put on how different family members’ emotions and behaviors reverberate off of one another to make moods spiral downwards or upwards. For example, “Child: Alone in bedroom → Parents: Worry and suggest alternative activities → Child: Becomes irritable and snaps at parents → Parents: Feel frustrated and withdraw → Child: Feels guilty and sad, withdraws more” (Tompson et al., 2020, p. 690). Over the course of the intervention, family members engage in multiple activities to improve family interaction patterns and a variety of family skills (e.g., family problem-solving). Although siblings are not systematically included in FFT-CD for every family, they are when clinically indicated (e.g., when sibling conflict is related to depressed mood; Tompson et al., 2020). In a randomized clinical trial comparing FFT-CD to individual supportive child therapy for depression, children in the FFT-CD group had higher levels of depression response and their families reported greater knowledge of depression management techniques than those in the individual therapy group (Tompson et al., 2017), although by the one-year follow-up assessment, children in the individual therapy group had caught up to the FFT-CD group (Asarnow et al., 2020).

Another approach that is centered around emotion regulation at the family level is Families Over-Coming Under Stress (FOCUS), a preventive intervention for individual families that have experienced a traumatic event or other significant adversity. FOCUS was designed with the whole family system, as well as multiple subsystems, in mind, so it is one of the few interventions that addresses family-level emotion regulation within all of the systems discussed in the current paper (caregiver–child, co-caregiver, sibling, and whole family systems). FOCUS for Families (Saltzman et al., 2011) may be used with families of children aged 5–18 years, while FOCUS for Early Childhood (Mogil et al., 2015) is specifically designed around the needs of preschool-aged children. Over the course of caregiver-only, child-only, and whole family sessions, caregivers and children are taught that a traumatic event – even when experienced by a single family member – reverberates throughout the entire family system. Families are taught to identify trauma reminders not only within themselves, but also to communicate about reminders among family members, and make a plan for coping with them together. For example, a family might identify that a fireworks display, while exciting for some family members, is a trauma reminder for a parent who experienced a combat deployment. The parent would share where they are on a feelings thermometer when the fireworks start, and children might be asked to describe how they can tell that their parent is feeling different from them (e.g., tense body, snaps, doesn’t want to be with the rest of the family). The family then develops a plan together for how to cope with the reminder; for example, they might develop a plan for coregulation, such that the non-deployed parent takes children to see the fireworks so that the children can still enjoy them, while the previously deployed partner is able to “tap out” and engage in healthy emotion regulation strategies to cope with the triggering event.

Consistent with many trauma-informed interventions, another central component of FOCUS is narrative work. However, unlike other interventions, the narrative component in FOCUS is not used for exposure, but rather, as a tool for emotional communication among family members (Saltzman et al., 2013). For families with school-aged or adolescent children, family members create individual visual narratives over the course of multiple parent-only, child-only, and whole family sessions; ultimately, the individual narratives are integrated into a family narrative. Each family member indicates on a timeline where they were on the feelings thermometer during significant events. A provider facilitates family members sharing their thoughts and feelings during each event, helping to identify and correct miscommunications or misunderstandings that may have occurred at times of major distress, and identify family strengths and resources that helped them cope. For families with preschool-aged children who are too young to create their own narratives, FOCUS-Early Childhood focuses on other, more developmentally appropriate techniques to enhance communication, including enhancing caregiver reflective functioning and play (Mogil et al., 2015). After creating their own narratives, caregivers are guided to put themselves in the minds of their child, and create a narrative from their child’s perspective. Caregivers revisit the major family events that they included on their own timelines, and imagine the emotions and thoughts that their child might have been experiencing during those times. This exercise is designed to enhance parents’ perspective taking and reflective functioning “muscle,” which should improve parent–child communication no matter the child’s developmental level. Play is conceptualized as a central mechanism through which children tell their stories, so significant time is spent teaching parents about the importance of play as well as key skills to enhance play, such as praise and reflection. Importantly, to enhance emotional communication with young children, parents are provided with psychoeducation about emotion socialization, and taught emotion coaching skills. Caregivers receive in vivo coaching of play and emotion coaching techniques during family sessions that are attended by the child and all participating caregivers.

Although the ultimate goal of the FOCUS narrative is to create a *family* narrative, there is intentionality in building that narrative incrementally. Caregivers first build their individual narratives in adult-only sessions; this allows opportunity for caregivers to reflect on how their own emotional experiences impacted the family system, and, importantly, in multiple caregiver families it highlights the importance of caregiver coregulation (including among more than two caregivers, when relevant; Garcia et al., 2017). For families with more than one child, the child-only sessions offer the opportunity for children to learn emotional content alongside their sibling, providing an opportunity to enhance sibling coregulation. FOCUS has not been tested against individual trauma treatments for children or adults, so the incremental value of family-level intervention for trauma/adversity above and beyond individual treatment is unknown. However, a randomized controlled trial of FOCUS-Early Childhood with military and veteran families indicated that those randomized to the intervention condition showed greater improvement on a variety of outcomes in comparison with families randomized to a web-based parenting education curriculum, including parental mental health symptoms (depression, anxiety, PTSD), child emotional and behavioral difficulties, family functioning (parent-reported), parenting behavior (observed and parent-reported), and children’s engagement with their parents (observed), which were found at 12-month follow-up (Mogil et al., 2021). These RCT findings were supportive of earlier program evaluation data from a large-scale implementation of FOCUS (*N* = 2615 families), which indicated improvements in a variety of parent and child mental health symptoms, child prosocial behavior, and family functioning (Lester et al., 2016).

Importantly, movement toward a more family-centered approach in promoting the development of children’s regulatory capacities does not necessarily involve re-imagining entire intervention programs and starting from scratch. Rather, there are ways in which current intervention approaches may be modified in terms of specific psychoeducation, skill-building, and therapeutic activities in order to engage the larger family system.

## Future Directions

### Greater Consideration of Positive Emotions

Although discussions of emotion regulation often reference both positive and negative emotions, there has been significantly more research exploring children’s capacity to modulate negative emotions (Lindsey, 2020). Shewark and Blandon (2015) have similarly noted the limited investigation of caregivers’ responses to children’s expressions of positive emotion and how such responses may contribute to the development of children’s regulatory capacities. This disproportionate focus is understandable as children’s difficulties regulating emotions such as anger, frustration, sadness, and anxiety can presage longer-term mental health issues and are stressful for parents and other adults to manage. The extant research, however, demonstrates that unmodulated positive emotion is related to externalizing behaviors whereas difficulty sustaining positive emotion is related to internalizing disorders; conversely, positive emotion of low intensity is associated with better self-regulation (Shewark & Blandon, 2015).

Fortunately, caregivers can play an important role in promoting and maintaining children’s positive affective states. Naturalistic observations, for example, have found that caregivers’ expressions of their own positive emotion, physical touch, and engagement sustained school-aged children’s expression of positive emotion (Bai et al., 2016). There is also an emerging focus on the link between humor and emotion regulation in both adults (Braniecka et al., 2019; Horn et al., 2019; Wu et al., 2021) and children and adolescents (Kuhlman et al., 2021; Mireault et al., 2015). A recent study found that infants aged five to seven months smiled more frequently, longer, and more quickly in response to an absurd event when caregivers provided cues that the event was humorous than when they provided neutral cues, suggesting caregivers can begin shaping their children’s understanding of humor very early on (Mireault et al., 2015). In another study, caregivers’ use of humor was associated with greater competence in emotion regulation among five- and six-year-old children, with parenting warmth mediating the relationship between maternal humor and children’s emotion regulation (Oh & Hwang, 2018). In a study of adolescents, self-reported use of humor appeared to have a protective effect against the impact of the COVID-19 pandemic on psychiatric symptoms (Kulman et al., 2021). In line with a strengths-based perspective, promoting caregivers’ use of coregulation strategies that also attend to positive emotions, including those that arise from humorous experiences, may not only benefit children’s individual development, but may also enhance the quality of interactions within various family subsystems. For example, caregivers may be more likely to alert to and potentially intervene when siblings are expressing anger toward one another, and less likely to notice when siblings are having fun together and their interactions are well regulated. There may be value in coaching caregivers to attend to and explicitly comment on the exchange of positive emotions in sibling relationships, which may help reinforce the sibling subsystem as an important part of the secure family base. Similarly, when coparents amplify the positive experiences they observe one another having with their child, it may serve as an important counterbalance to the more difficult moments of parenting.

### Broader Inclusion of Diverse Family Constellations

Research efforts focused on understanding emotion regulation and coregulation as family-level phenomena would be well served by paying greater attention to the increasing diversity and complexity of family systems. Not all families are led by a mother and father, or for that matter by two caregivers who also have an intimate relationship with one another and reside in the same home. Many families are headed by gay and lesbian caregivers, single caregivers, two caregivers–one of whom resides elsewhere, by a parent and grandparent, or by multiple family members who function in important caregiving roles. A slowly emerging body of work exploring these different family constellations has documented linkages between emotion regulation and dysregulation across different family subsystems. For example, in a study of adolescent mothers of Mexican origin (Derlan et al., 2017) found that conflicts about parenting in the mother-grandmother relationship were associated with conflicts about parenting in the mother-father relationship. In another recent study (Salman-Engin et al., 2018), observations of triadic interactions among families in Turkey found that mothers’ behavior toward their infants varied depending on whether grandmothers or fathers were participating in the interaction. Despite these advances, such research is not specifically focused on emotion regulation and coregulation per se, and future work would help elucidate the ways in which adults and children navigate their emotional lives in these family contexts.

Importantly, there is not only increasing diversity across families, but within families as well. There is scant information about what happens in families when coparents bring together a range of culturally based beliefs about how emotions should be expressed and managed within and outside the family, including beliefs that may be rooted in a desire to protect children and families from discrimination, bias, and trauma, as discussed earlier. When caregivers bring vastly different personal experiences into their newly formed families, they are faced with the task of finding a way to establish emotion socializations practices as a coparenting team that takes these differences into account. Understanding how caregivers are able to successfully navigate this process would help inform prevention and intervention efforts to support the needs of increasingly diverse families.

### Role of Moderating Factors

The patterns of emotion regulation and coregulation that occur within and across family subsystems and at the whole family level are likely influenced by the individual characteristics of family members, including the child’s age and gender, as well as the caregiver’s gender. As noted earlier, children’s regulatory capacities and need for coregulation undergo significant changes across the developmental course. Caregivers may need to transition away from the emotional and physical comforting and direct coaching that occur in early childhood to supporting their older children’s and adolescents’ efforts to identify and utilize emotion regulation strategies more independently (Houltberg et al., 2012). Brand et al., (2010) has observed that “these emotionally evocative developmental tasks potentially tax both the adolescent’s and his or her parents’ abilities to regulate the strong emotions that typically accompany adolescence” (p. 87). Van Lissa et al. (2019) have further noted that caregivers may have higher expectations for their adolescent’s ability to manage their emotions and if those expectations aren’t met, caregivers may intervene with more supervision and control. Indeed, the more intense emotions that can accompany adolescence (Reitsema et al., 2021) may pull caregivers in at the very time that their teen is pushing away which may lead to a higher level of negative emotions throughout the family system.

One question that arises is how the adolescent’s changing needs for coregulation are navigated at the family level. Different caregivers in the family may be differentially affected by these changing needs–for example, if one caregiver has historically been more directly involved in their child’s emotional life, they may be the caregiver who the adolescent is pushing away to a greater degree and who may feel more “rejected” by the child. This may call for a shift in roles in which the other caregiver steps up to provide the teen with needed guidance and guardrails so the other caregiver can step back. While this caregiver is now more explicitly involved in responding to their adolescent’s day-to-day emotional needs, this change has been made possible by reorganizing coregulation at the level of the entire family system in order to provide an environment in which the adolescent’s emotional development and need for increased autonomy can both be supported. Although the trajectory of emotion regulation during childhood and adolescence has been the focus of a number of researchers (Morris et al., 2017; Reitsema et al., 2021; Silvers, 2022), there remains a need to understand how patterns of emotion regulation and coregulation shift within and across different subsystems and the whole family system as children in the family progress through different developmental stages.

A number of studies have also examined differences in how boys and girls experience and express emotions, as well as differences in how mothers and fathers respond to children’s need for coregulation. For example, one study found that girls display more positive emotions than boys, as well as more internalizing emotions; in contrast, boys initially showed more externalizing emotions than girls, although by adolescence girls were displaying more externalizing emotions (Chaplin & Aldao, 2013). These different patterns of emotional expressiveness have been partially attributed to differences in temperament between boys and girls, as well as differences in how caregivers socialize emotion in boys versus girls (Root & Denham, 2010; Root & Rubin, 2010). Moreover, caregivers may react differently to temperamental variations in boys and girls; for example, Grady (2020) found that caregivers reported they were more likely to respond in a minimizing fashion to negative emotions in shy boys than in shy girls. There is also evidence that the gender of the caregiver plays a role in coregulation practices (Denham et al., 2010), and different patterns also emerge across same-gender and opposite-gender caregiver–child dyads (Feldman, 2003). Understanding different patterns of emotion regulation and coregulation within and across family subsystems and at the whole family level becomes rapidly more complex when considering both child and caregiver gender, especially when there are multiple children in the family. Avenues for future exploration might be to examine the coregulation strategies that mothers and fathers use in the context of triadic or whole family interactions, whether there are reliable patterns as a function of caregiver gender, and furthermore, whether such patterns are evident across opposite-gender (mother/father) and same-gender (mother/mother or father/father) coparenting teams.

### Methodological Considerations

Measuring family-level constructs is challenging for a number of reasons. Self-report questionnaires are likely the most widely used data collection technique in the social sciences, but a major challenge of family research is that the construct of interest—whether dyadic, triadic, or more—cannot report on itself. Some studies collect data from multiple family members and then aggregate across them (Krug et al., 2016), which is theoretically more precise (Georgiades et al., 2008) than studies that use the perspective of one family member as the representative source (Baxter et al., 2011; Jellett et al., 2015). However, in many cases, discrepancies in family members’ reports appears to be systematic, and therefore averaging these data blur what could be important information (Cook & Goldstein, 1993; Jacob & Windle, 1999). For example, adolescents and pre-adolescents tend to reported worse family functioning than their parents (Human et al., 2016; Leung et al., 2016; Ohannessian et al., 2000, 2016), and children tend to report lower levels of parental acceptance than their mothers and fathers, and higher levels of parental psychological control than their mothers (Korelitz & Garber, 2016). A major challenge in reliably measuring family relationships and functioning is capturing the overall family’s perspective using responses provided by individual family members, whose reports may vary due to different roles within the family. This is even more challenging to do if a family system includes children who are too young to self-report, or households in which multiple languages are spoken that are not all represented by the same self-report measure.

Interview approaches offer another avenue to advance our understanding of how caregivers’ self-regulatory capacities and coregulation practices can impact the development of children’s emotion regulation skills through self-report. The Meta-Emotion Interview (Katz & Gottman, 1986) assesses parental meta-emotion philosophy (PMEP) or what has been described as “an organized set of feelings and thoughts that parents have about their own emotions and those of their children” (p. 47, Katz et al., 2012), including their beliefs about the importance of discussing, validating, and problem-solving around their children’s negative emotions (emotion coaching) versus minimizing or ignoring negative emotions in order to eliminate them as quickly as possible (emotion dismissing). Various dimensions of PMEP have been linked to indicators of children’s emotion regulation. For example, adolescents’ perceptions (as assessed by the Meta-Emotion Interview) of low parental emotion coaching in regard to sadness predicted their onset of major depressive disorder (Schwartz et al., 2018). Another interview methodology, The Parent Development Interview (Slade et al., 1999) assesses parental reflective functioning (PRF), or the caregiver's capacity to recognize that their own internal mental states, including affective experiences, are different from those of their child, to observe and reflect on their child’s inner experiences, and to share those reflections with their child in a way that enhances the child’s understanding of their own emotional life. Better maternal reflective functioning has been associated with infants’ ability to self-soothe during a still face procedure (Heron-Delaney et al., 2016) and young children’s ability to regulate negative emotions during a frustrating task (Dollberg et al., 2009). Although these interviews are administered individually, utilizing multiple family members’ responses to create profiles of dyads (e.g., coparents, caregiver–child) or triads (e.g., coparents and adolescent) could provide a window into caregivers’ respective reflections on how they should or do manage their children’s emotions. Creating such profiles may reveal how, taken together, caregiver beliefs or reflective capacities complement, buffer, or positively or negatively amplify one another either to the benefit or detriment of their children’s emotional development.

Given the challenges of self-report measures on multi-person constructs, observational methods are widely used in family research as a way to capture interaction within dyads (including parent–child, Mogil et al., 2021; co-caregiver, Mammen et al., 2016; siblings, Kramer & Gottman, 1992) or larger family units (Hollenstein et al., 2016; McHale et al., 2008; Paley et al., 2005). In a study discussed earlier, Lunkenheimer et al. (2012) noted that the challenges of measuring family interactions have limited our understanding of how moment-to-moment emotional transactions within families shape children’s development of emotion regulation skills. These researchers have used state space grids of family discussions to examine how flexibility or variability in family members’ emotion socialization practices relate to children’s emotion regulation. This approach entails mapping interpersonal behaviors onto a two-dimensional grid, in this case different emotion socialization functions (*x*-axis) and emotion words (*y*-axis), with the goal of capturing how diversified the family’s repertoire is for navigating emotional experiences. The authors suggest that greater flexibility may reflect the family’s ability to quickly adapt to shifting interpersonal dynamics, and may allow the child to develop a wider range of strategies for responding effectively to emotionally evocative challenges. Another recent study of triadic interactions used state space grids to explore triadic affective patterns in families with and without depressed adolescents, finding that families with depressed adolescents exhibited less predictability in their affective sequences, stayed longer and returned more quickly to discrepant affective states, and spent less time and took longer to return to matched affective states (Hollenstein et al., 2016). Importantly, this approach allows for the triad to be examined as a whole system rather than as a combination of three dyads (Hollenstein et al., 2016). However, while observational methods offer rich information on family interactions, maximizing the data requires complex coding and analytic approaches and thus are generally more time-consuming for researchers. Additionally, because observations of family interactions typically require some sort of task standardization and often occur in a lab setting with materials (e.g., toys) that might not be familiar to the family, there may be some issues with ecological validity of this type of methodology.

Experience sampling method (ESM; Hektner et al., 2007) and related approaches hold promise as a method for future research on emotion regulation at the family level, partly because they address several limitations of other common methodologies. ESM and related approaches are characterized by (a) *repeated assessments* of (b) *current* or *very recent states* (c) in the context of individual participants’ *natural environments* (Trull & Ebner-Priemer, 2009). Most modern ESM studies involve participants being contacted by the researcher periodically throughout the day or week and prompted to answer questions on a mobile device. There are many benefits of ESM beyond traditional self-report rating scales and interviews, including that being asked to report on current or very recent events (e.g., how sad a participant felt in the 5 min before receiving the study ping) reduces memory biases that arise when participants are asked via questionnaire or interview to report on past events, or to summarize phenomena that are ongoing and dynamic (e.g., when asked to report on depressed mood over the past 2 weeks). ESM has been used not only with adults, but also with elementary school-aged children (Benoit Allen et al., 2016; Whalen et al., 2006) and adolescents (Silk et al., 2011). Thus, ESM could be used to capture emotion across multiple individuals in a family system without the need for travel to a laboratory- or home-based observational task or time-consuming microsocial coding of interactions. Furthermore, ESM may be more ecologically valid than laboratory observations because it is more likely to obtain assessments in contexts that elicit the full range of various family member emotions, it does not rely on situations that may seem artificially contrived, and it allows states to be examined beyond a relatively short time frame (Trull & Ebner-Priemer, 2009).

Although not focused on emotion regulation specifically, a recent study elegantly captured how multiple family members’ reports on ESM can be captured and analyzed to shed light on family-level transfer of stress and conflict (Timmons et al., 2017). In a study of 114 families, mothers and fathers provided 2 weeks worth of individual daily reports on levels of marital conflict and a variety of stressors. Among many interesting results, the authors found that marital conflict was highest on days when both partners experienced high stress. However, they found that the relation between marital conflict and stress level was particularly dependent on wives’ daily stress. Specifically, there was a strong relation between wives’ reports of conflict and stress levels no matter the level of stress reported by their partners, while husbands’ stress was only significantly related to conflict if their partner experienced high stress (versus low stress) on that day (Timmons et al., 2017). Future ESM studies could assess emotional states, coping strategies, and a variety of family interaction variables across family members, providing an opportunity to examine emotion-related reports of multiple family members in their natural environment.

## Conclusion

The capacity to effectively navigate one’s emotional experiences–both positive and negative– is well established as a critical building block for healthy development. Moreover, there is ample evidence that supportive relationships can be a powerful conduit by which to promote children’s development in this domain. Traditionally, the caregiver–child dyad has been the focal point of much of the research on coregulation, with numerous studies identifying the features of such dyadic interactions that give rise to adaptive emotion regulation skills. Such studies provide compelling evidence for the ways in which a caregiver can play a pivotal role in helping children learn to manage their emotions. However, a growing body of family-centered research illustrates that widening the lens to consider the ways in which the larger family system shapes and is shaped by this foundational developmental process also identifies points of entry for prevention and intervention approaches that support the establishment of healthy patterns of emotion regulation and coregulation for all family members.

## Data Availability

Not applicable.

## Footnotes

1 The term “caregiver” will be used in this paper to be inclusive of all adults who function in a parenting role.
